# Neutrophil extracellular trap formation is increased in psoriasis and induces human β-defensin-2 production in epidermal keratinocytes

**DOI:** 10.1038/srep31119

**Published:** 2016-08-05

**Authors:** Stephen Chu-Sung Hu, Hsin-Su Yu, Feng-Lin Yen, Chi-Ling Lin, Gwo-Shing Chen, Cheng-Che E. Lan

**Affiliations:** 1 Department of Dermatology, College of Medicine, Kaohsiung Medical University, Kaohsiung, Taiwan; 2 Department of Dermatology, Kaohsiung Medical University Hospital, Kaohsiung, Taiwan; 3 National Institute of Environmental Health Sciences, National Health Research Institutes, Taipei, Taiwan; 4 Department of Fragrance and Cosmetic Science, College of Pharmacy, Kaohsiung Medical University, Kaohsiung, Taiwan; 5 Department of Dermatology, Kaohsiung Municipal Hsiao-Kang Hospital, Kaohsiung, Taiwan

## Abstract

Neutrophil extracellular traps (NETs) have been implicated in the development of certain immune-mediated diseases, but their role in psoriasis has not been clearly defined. Human β-defensin-2 (HBD-2) is an important antimicrobial peptide overexpressed in psoriasis epidermis. We evaluated whether the amount of NETs is increased in psoriasis and determined the effect of NETs on HBD-2 production in epidermal keratinocytes. Using fluorescent microscopy, we found that patients with psoriasis (n = 48) had higher amount of NETotic cells in their peripheral blood compared to healthy controls (n = 48) and patients with eczema (n = 35). Psoriasis sera showed increased ability to induce NET formation in control neutrophils but normal NET degradation ability. The amount of NETs in the peripheral blood correlated with psoriasis disease severity. NETosis was also observed in the majority (18 of 20) of psoriasis skin specimens. Furthermore, NETs induced HBD-2 mRNA and protein production in keratinocytes, and immunohistochemical analysis confirmed strong expression of HBD-2 in psoriasis lesional skin. In summary, NET formation is increased in peripheral blood and lesional skin of psoriasis patients and correlates with disease severity. Additionally, NET-induced HBD-2 production may provide a novel mechanism for the decreased susceptibility of psoriasis plaques to microbial infections.

Psoriasis is a common chronic immune-mediated disease and is clinically characterized by well-demarcated erythematous plaques with white or silvery scales which can involve any part of the skin^1^. In addition to skin involvement, a subset of patients may suffer from psoriatic arthritis with joint pains and deformities. Psoriasis can be a severe disease which affects a patient’s ability to function and causes substantial impairment to quality of life^2^. Recent research has also shown that psoriasis may be associated with various systemic disorders, including diabetes mellitus^3^, metabolic syndrome^4^, vascular complications (such as stroke and ischemic heart disease)^5^,^6^,^7^, and depression^8^. Currently, although various investigations have suggested important roles for Th1 and Th17 cells^9^, the etiology and pathogenesis of this disease remain incompletely defined.

Various studies have highlighted the role of the innate immune system in the pathogenesis of psoriasis^10^,^11^,^12^. Neutrophils are recruited to psoriasis lesions, particularly in the epidermis where they cluster to form spongiform pustules of Kogoj in the stratum spinosum and Munro’s microabscesses in the stratum corneum^13^. Although the involvement of neutrophils in the pathogenesis of psoriasis is currently not understood, a critical role is suggested by reports of psoriasis improvement during drug-induced agranulocytosis and its recurrence after the normalization of neutrophil counts^14^. Fumaric acid derivatives, which are increasingly used to treat psoriasis^15^, have also recently been found to inhibit neutrophil functions^16^. Furthermore, recent studies showed that neutrophils express IL-17 in psoriasis skin lesions, suggesting that these cells may play a role in psoriasis pathogenesis^17^,^18^.

Neutrophils have been demonstrated to form neutrophil extracellular traps (NETs), which bind and destroy microbial pathogens in the extracellular space^19^. NETs are web-like structures which consist of decondensed DNA associated with histones and antimicrobial peptides^20^,^21^,^22^,^23^. They are derived from the granular and nuclear contents of neutrophils. The release of NETs results in a high local concentration of antimicrobial peptides, including myeloperoxidase, neutrophil elastase and cathelicidin LL37. During the process of NETosis, the nuclear and granule membranes dissolve, the nuclear DNA/histones unfold and bind to granular proteins, which are then released into the extracellular space. NETs are mainly degraded by the action of the DNA degrading enzyme, deoxyribonuclease I (DNase I)^24^. Although NETs are beneficial in antimicrobial defense, they may act as a source of autoantigens, and have been implicated in the development of autoimmune diseases such as small vessel vasculitis^25^, systemic lupus erythematosus (SLE)^24^,^26^,^27^,^28^,^29^, and rheumatoid arthritis^30^. However, the role of NETosis in the pathogenesis of psoriasis has not been clearly defined previously.

It is well known that psoriasis patients have a low incidence of cutaneous infections^31^. This may be explained by the increased expression of various antimicrobial peptides in psoriasis skin lesions^32^. In particular, human β-defensin-2 (HBD-2) is highly-expressed in psoriasis plaques and has been found to be the most psoriasis-specific antimicrobial peptide^33^,^34^,^35^. In contrast, HBD-2 is expressed at negligible or low levels in normal skin and skin lesions of eczema^33^,^34^,^35^,^36^. Currently, the molecular mechanisms for increased HBD-2 expression in psoriatic plaques are not well defined.

In this study, we examined whether the amount of NETotic cells in the peripheral blood is increased in patients with psoriasis compared to healthy controls and patients with eczema, and whether the level of NETosis correlates with disease severity. We also explored whether psoriasis sera have altered ability to induce NET formation and NET degradation in control neutrophils. In addition, we evaluated whether NETosis can be observed in lesional skin of patients with psoriasis. Furthermore, we seek to determine whether NETs from psoriasis patients can induce HBD-2 expression in epidermal keratinocytes. The findings of this study provided new insights into the significance of NETosis in the pathophysiology of psoriasis, particularly the role of NETosis in the antimicrobial defense mechanisms of this disease.

## Results

### Clinical features of patients

In this study, we analyzed peripheral blood neutrophils and sera from 48 patients with psoriasis. The mean age of the psoriasis patients was 47.56 ± 15.41 years. There were 28 males and 20 females (Table 1). The clinical subtype was psoriasis vulgaris in 44 cases, guttate psoriasis in 2 cases, and erythrodermic psoriasis in 2 cases. Six patients had a history of psoriatic arthritis. The Psoriasis Area and Severity Index (PASI) score was <10 in 10 cases, 10–20 in 15 cases, 20–30 in 18 cases, and 30–40 in 5 cases. The treatments for patients with psoriasis included no treatment at the time of blood sampling (7 patients), topical therapy (34 patients; including topical steroids, vitamin D analogues, and retinoids), ultraviolet light phototherapy (28 patients), methotrexate (6 patients), cyclosporine (4 patients), and biological agent (ustekinumab; 3 patients).

We also included 48 healthy controls in this study, who were individually matched in terms of age and sex to the psoriasis patients (mean age 46.79 ± 15.11 years, 28 males and 20 females). There was no statistically significant difference in age between psoriasis patients and healthy controls (*P* = 0.805). In addition, we enrolled 35 patients with eczema (contact dermatitis, 10 patients; atopic dermatitis, 14 patients; general eczema, 11 patients) with >10% body surface area involvement for comparison (mean age 52.43 ± 20.89 years, 24 males and 11 females).

### Patients with psoriasis had higher amount of NETotic cells in their peripheral blood compared to healthy controls

Neutrophils are known to be recruited to psoriasis skin lesions and may be found in increased numbers in the peripheral blood in severe forms of the disease. Recently, increased numbers of NETotic cells have been found in the peripheral blood of patients with immune-mediated diseases such as SLE and rheumatoid arthritis^26^,^30^. However, similar studies have not been performed in psoriasis patients. We therefore set out to determine whether the amount of NETotic cells in the peripheral blood is increased in patients with psoriasis compared to healthy controls.

**Figure 1:**
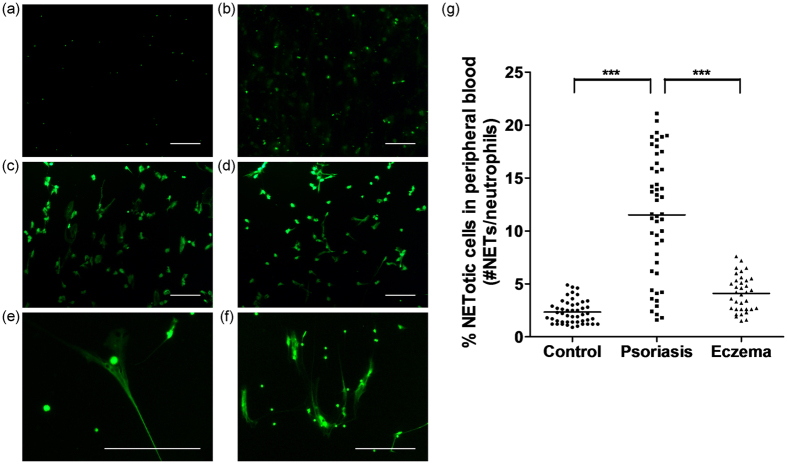
Evaluation of the amount of NETotic cells in the peripheral blood by fluorescence microscopy. **(a–f)** Images showing that the amount of NETotic cells in the peripheral blood is low in healthy controls (**a**) and high in patients with psoriasis (**b–d**). NETotic cells are characterized by nuclear expansion and extracellular web-like DNA fibers, as demonstrated under high-power views (**e**,**f**). Peripheral blood neutrophils undergoing NETosis were stained with the fluorescent extracellular DNA dye Sytox Green. Scale bars = 200 μm. **(g)** Patients with psoriasis had higher amount of NETotic cells in their peripheral blood compared to healthy controls and patients with eczema. Peripheral blood neutrophils undergoing NETosis were stained with the fluorescent extracellular DNA dye Sytox Green, and visualized using fluorescence microscopy. The percentage of NETotic cells was determined by dividing the number of cells undergoing NETosis by the total number of neutrophils. Each dot corresponds to one individual donor (psoriasis patients, n = 48; healthy controls, n = 48; eczema patients, n = 35). The horizontal lines denote the mean of the group. ***P < 0.001, ANOVA with Bonferroni correction.

Peripheral blood neutrophils undergoing NETosis were stained with the fluorescent extracellular DNA dye Sytox Green, and visualized using fluorescence microscopy. The morphology of peripheral blood neutrophils undergoing NETosis was characterized by nuclear expansion and extracellular web-like DNA strands (Fig. 1a–f). The percentage of NETotic cells was determined by dividing the number of cells undergoing NETosis by the total number of neutrophils. As shown in Fig. 1g, healthy controls had low amount of NETotic cells in their peripheral blood (2.33 ± 1.09%; minimum 0.9%, maximum 4.9%), while patients with eczema had slightly higher amount of NETs (4.10 ± 1.68%; minimum 1.5%, maximum 7.6%). The amounts of NETotic cells in the peripheral blood for different forms of eczema were as follows: 3.47 ± 1.33% (contact dermatitis, 10 patients), 4.83 ± 1.93% (atopic dermatitis, 14 patients) and 3.75 ± 1.37% (general eczema, 11 patients). There were no statistically significant differences in peripheral blood NET amount between the three different forms of eczema. In particular, patients with psoriasis had much higher percentage of NETotic cells in their peripheral blood (11.53 ± 5.77%; minimum 1.6%, maximum 21.1%) compared with healthy controls and patients with eczema.

### Sera from patients with psoriasis showed increased ability to induce NET formation in control neutrophils

The increased amount of NETotic cells in the peripheral blood of patients with psoriasis may be a result of two possible factors: 1) increased NET formation, or 2) impaired NET degradation. Firstly, we evaluated whether sera from psoriasis patients have increased ability to induce NET formation in control neutrophils.

**Figure 2:**
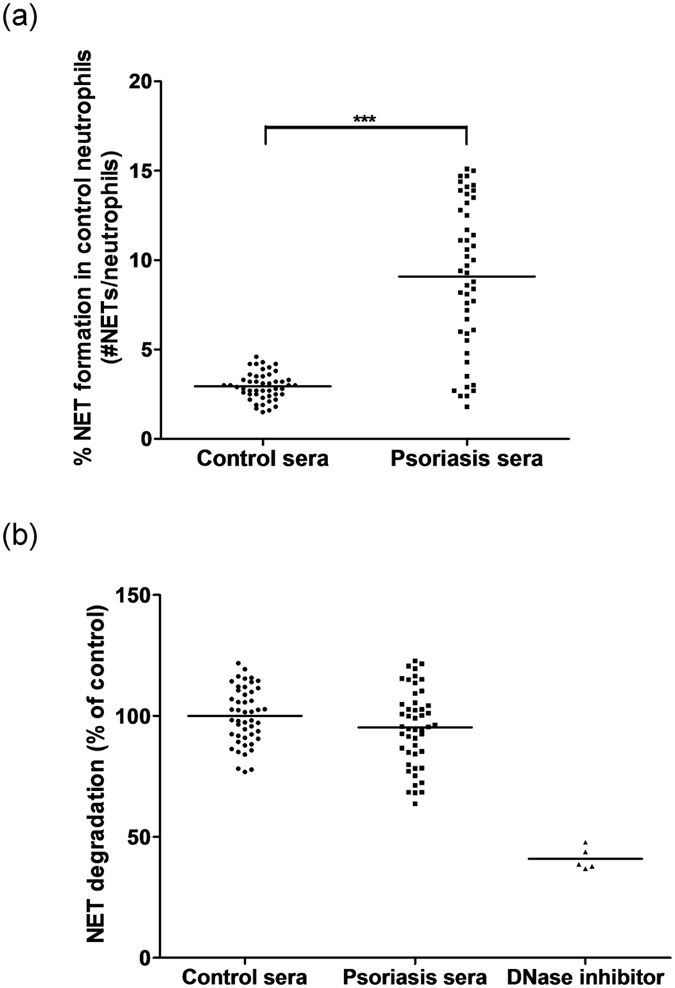
Effects of psoriasis sera on NET formation and NET degradation in control neutrophils. (**a**) Sera from patients with psoriasis (n = 48) showed increased ability to induce NET formation in control neutrophils compared to sera from healthy controls (n = 48). % NET formation in control neutrophils was determined by dividing the number of neutrophils forming NETs by the total number of neutrophils. Each dot corresponds to serum from one individual donor. The horizontal lines denote the mean of the group. ***P < 0.001, two sample t-test. (**b**) Sera from patients with psoriasis (n = 48) showed similar ability to degrade NETs from control patients compared to sera from healthy controls (n = 48). Control sera treated with DNase I inhibitor G-actin (n = 5) was included as a positive control. The amount of NET degradation by the sera of healthy controls was regarded as 100%. Each dot corresponds to serum from one individual donor. The horizontal lines indicate the mean of the group.

We incubated serum samples from 48 psoriasis patients and 48 healthy controls with unstimulated control neutrophils. Cells undergoing NETosis were stained with Sytox Green, and quantified by fluorescence microscopy. As shown in Fig. 2a, sera from healthy controls had low capacity to induce NET formation in control neutrophils (2.94 ± 0.76%). In comparison, sera from psoriasis patients showed increased ability to induce NET formation in control neutrophils (9.09 ± 4.13%).

### Sera from patients with psoriasis showed normal NET degradation ability

Previously, it had been demonstrated that sera from SLE patients had impaired ability to degrade NETs^24^,^29^. However, it is unknown whether psoriasis patients are characterized by diminished NET clearance. We therefore proceed to test the ability of psoriasis sera to degrade NETs from control patients.

Control neutrophils were stimulated to induce NETosis using 25 nM phorbol 12-myristate 13-acetate (PMA). Wells containing NETs were then incubated with 10% serum from psoriasis patients or healthy controls for 6 hours. The cell-free culture supernatants were collected, the fluorescent DNA dye PicoGreen was added, and the DNA content was assessed using a fluorescence spectrophotometer. As shown in Fig. 2b, sera from psoriasis patients showed similar NET degradation ability compared to sera from healthy normal controls. Therefore, NET degradation was not impaired in patients with psoriasis. As expected, treatment of control sera with DNase I inhibitor (10 μM G-actin) resulted in decreased NET degradation.

### Relationship between amount of NETotic cells in the peripheral blood and clinical parameters in psoriasis patients

We next investigated whether the amount of NETotic cells in the peripheral blood of psoriasis patients is associated with various clinical parameters (Table 1). Patients with psoriasis were categorized into the low NET amount group (n = 23) or high NET amount group (n = 25) using the mean amount of NETotic cells in the peripheral blood (11.53%) as the dividing point. We found that there was a positive association between NET amount and psoriasis severity (determined by PASI score) (*P* < 0.001). On the other hand, there was no statistically significant association between the amount of NETotic cells in the peripheral blood and patient’s age, sex, clinical subtype, presence of psoriatic arthritis, or treatment.

### Correlation between NETosis and psoriasis disease severity

**Figure 3:**
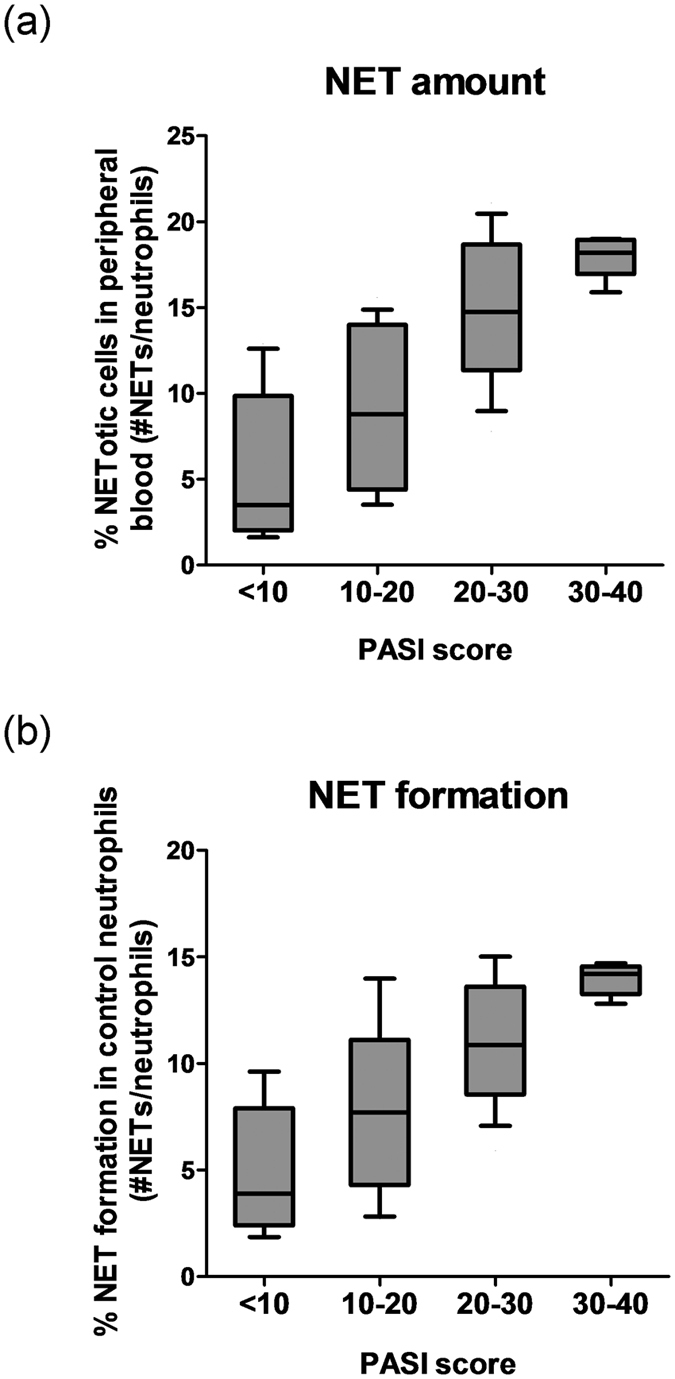
Correlation between NETosis and psoriasis disease severity. (**a**) Box-and-whisker plots (median with quartiles) showing the relationship between psoriasis clinical severity (as determined by PASI score) and amount of NETotic cells in the peripheral blood in patients with psoriasis. The percentage of NETotic cells was determined by dividing the number of cells undergoing NETosis by the total number of neutrophils. (**b**) Box-and-whisker plots showing the relationship between psoriasis clinical severity (PASI score) and induction of NET formation in control neutrophils by psoriasis sera. (PASI < 10, 10 patients; PASI 10–20, 15 patients; PASI 20–30, 18 patients; PASI 30–40, 5 patients).

We next proceed to further evaluate the correlation between NETosis and the clinical severity of psoriasis, as determined by PASI score. We found that there was a positive correlation between the amount of NETotic cells in the peripheral blood and psoriasis severity (correlation coefficient 0.604; *P* < 0.001). The mean percentage of NETotic cells in the peripheral blood of psoriasis patients with different degrees of disease severity were as follows: 5.72 ± 4.33% (PASI < 10), 9.35 ± 4.60% (PASI 10–20), 14.77 ± 4.10% (PASI 20–30), 18.00 ± 1.25% (PASI 30–40) (Fig. 3a).

In addition, there was a statistically significant correlation between induction of NET formation in control neutrophils by psoriasis sera and disease severity (correlation coefficient 0.575; *P* < 0.001). The mean NET formation in control neutrophils exposed to sera of psoriasis patients with different degrees of disease severity were as follows: 4.87 ± 2.91% (PASI < 10), 8.04 ± 3.93% (PASI 10–20), 10.96 ± 2.77% (PASI 20–30), 13.96 ± 0.74% (PASI 30–40) (Fig. 3b).

### Lesional skin from psoriasis patients showed increased amount of NETs

It had been previously demonstrated that in patients with autoimmune small vessel vasculitis, SLE and rheumatoid arthritis, neutrophils undergoing NETosis could be found in peripheral tissues, including the skin and kidneys^25^,^28^,^30^. Having identified that patients with psoriasis had increased amount of NETs in their peripheral blood, we next evaluated whether psoriasis skin lesions are infiltrated by neutrophils undergoing NETosis.

**Figure 4:**
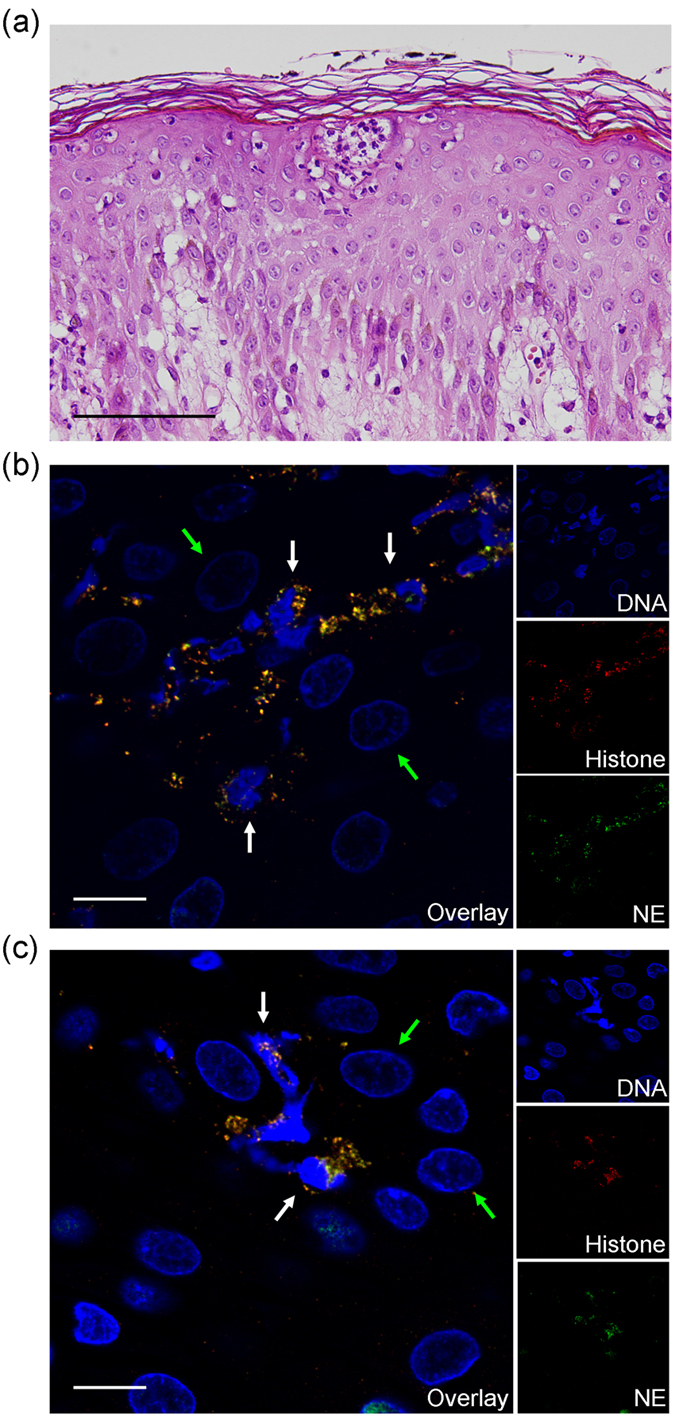
Neutrophils undergoing NETosis are frequently observed in the epidermis of psoriatic plaques. (**a**) Histology of psoriasis skin lesion (stained with hematoxylin and eosin) demonstrating neutrophil aggregation in the epidermis. Scale bar = 200 μm. (**b**,**c**) Confocal microscopic images of psoriasis skin tissue specimens. NETs (white arrows) are frequently seen in the epidermis next to keratinocytes (green arrows). Neutrophils undergoing NETosis are demonstrated as strandlike DNA structures (DAPI, blue) with concurrent dot-like staining for histones (red) and neutrophil elastase (NE, green). Scale bars = 10 μm.

Twenty cases of psoriasis skin tissue specimens and 20 cases of eczema skin specimens were stained for DNA (DAPI), histones (anti-H2A), and a neutrophil granule marker (anti-neutrophil elastase). Slides were analyzed using a confocal microscope. NETs were identified as extracellular strandlike DNA structures, with concurrent dot-like staining for histones and neutrophil elastase. We identified neutrophils undergoing NETosis in the majority of psoriasis skin specimens (18 out of 20 cases), particularly in the epidermis (Fig. 4). It should be noted that the two negative cases without visible NETosis belong to psoriasis patients with less severe skin disease (PASI score < 8) and low amount of NETotic cells in the peripheral blood (<5%). In contrast, NETs were not seen in lesional skin of 20 patients with eczema (Supplementary Figure 1).

### Increased expression of HBD-2 in psoriasis skin lesions

It is well known that psoriasis patients have a low incidence of cutaneous infections^31^. This has been attributed to the increased expression of various antimicrobial peptides in psoriasis skin lesions^32^. In particular, HBD-2 is highly expressed in psoriasis plaques and has been found to be the most psoriasis-specific antimicrobial peptide^33^,^34^,^35^.

**Figure 5:**
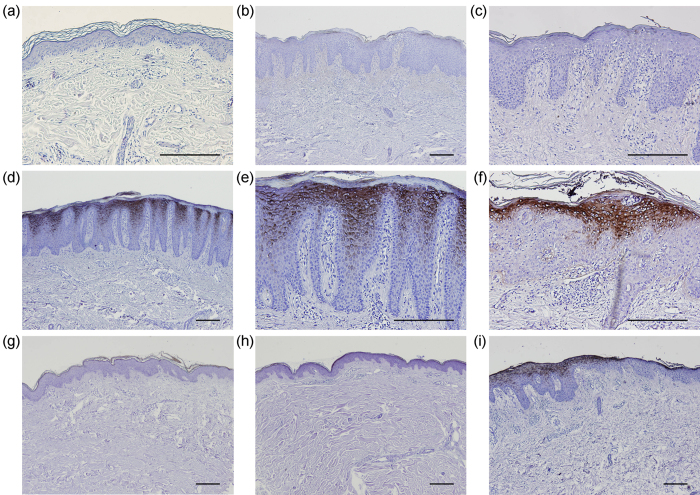
High expression of HBD-2 in the epidermis of psoriasis skin lesions. Immunohistochemical staining showed negative or weak expression of HBD-2 peptide in normal skin (**a**) and eczema skin lesions (**b**,**c**). In contrast, strong expression of HBD-2 was found in psoriasis lesional skin, localized to the upper epidermis and stratum corneum (**d–f**). In addition, weak HBD-2 expression was demonstrated in psoriasis nonlesional skin (**g**,**h**). A clear transition from strong to weak HBD-2 expression was seen in the junction between psoriasis lesional and perilesional skin (**i**). Scale bars = 500 μm.

Twenty-eight cases of psoriasis lesional skin tissue specimens, 10 cases of psoriasis nonlesional skin, 15 cases of eczema skin (general eczema), and 10 cases of normal skin were stained for HBD-2. Immunohistochemical staining showed negative or weak expression of HBD-2 peptide in all cases of normal skin (Fig. 5a) and eczema skin lesions (Fig. 5b,c). In contrast, strong expression of HBD-2 peptide was found in 26 out of 28 (93%) cases of psoriasis lesional skin, localized to the upper epidermis and stratum corneum (Fig. 5d–f). The two psoriasis cases with weak HBD-2 expression were the same as the two cases which showed no observable NETs in lesional skin. In addition, weak HBD-2 expression was demonstrated in all cases of psoriasis nonlesional skin (Fig. 5g,h). A clear transition from strong to weak HBD-2 expression was seen in the junction between psoriasis lesional and perilesional skin (Fig. 5i).

### Netting neutrophils from psoriasis patients induced HBD-2 mRNA expression in epidermal keratinocytes

**Figure 6:**
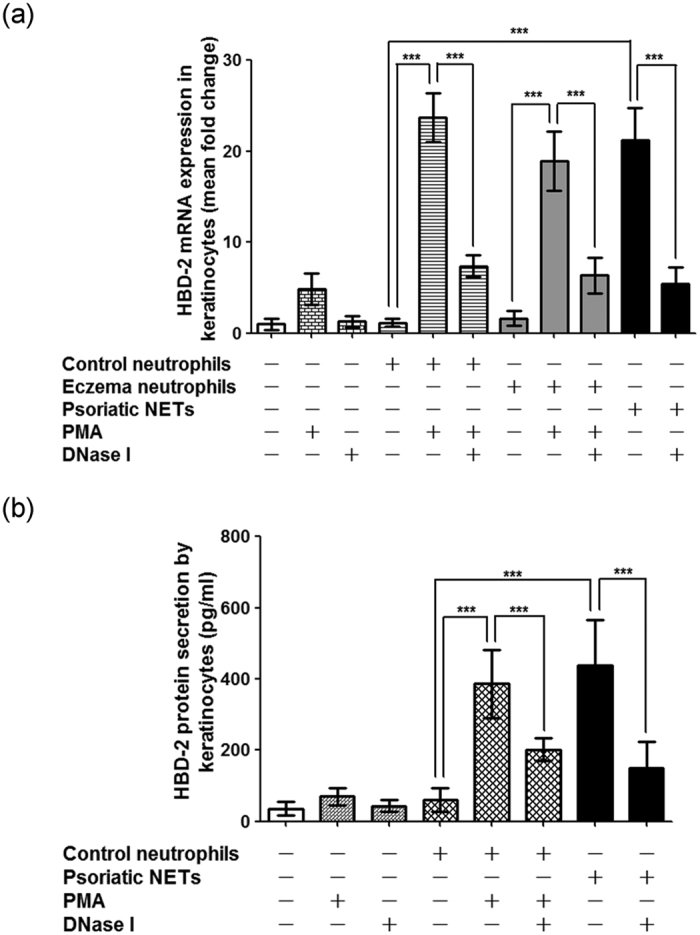
Netting neutrophils from psoriasis patients induced HBD-2 mRNA and protein expression in epidermal keratinocytes. (**a**) Normal human keratinocytes were obtained from adult foreskin, and peripheral blood neutrophils were isolated from 11 healthy controls, 7 eczema patients and 9 psoriasis patients. Normal human keratinocytes were treated with PMA or DNase I alone, or co-cultured for 16 hours with unstimulated control neutrophils (n = 11), control neutrophils stimulated with PMA (n = 11), control neutrophils treated with both PMA and DNase I (n = 11), unstimulated eczema neutrophils (n = 7), eczema neutrophils stimulated with PMA (n = 7), eczema neutrophils treated with both PMA and DNase I (n = 7), psoriatic neutrophils (n = 9), and psoriatic neutrophils treated with DNase I (n = 9). HBD-2 mRNA expression from keratinocytes was determined by real-time quantitative RT-PCR. ***P < 0.001, ANOVA with Bonferroni correction. (**b**) Netting neutrophils from psoriasis patients induced HBD-2 protein secretion by epidermal keratinocytes. Normal human keratinocytes were obtained from adult foreskin, and peripheral blood neutrophils were isolated from 7 healthy controls and 7 psoriasis patients. Normal human keratinocytes were treated with PMA or DNase I alone, or co-cultured for 24 hours with unstimulated control neutrophils (n = 7), control neutrophils stimulated with PMA (n = 7), control neutrophils treated with both PMA and DNase I (n = 7), psoriatic neutrophils (n = 7), and psoriatic neutrophils treated with DNase I (n = 7). HBD-2 protein secretion from keratinocytes was determined by ELISA. ***P < 0.001, ANOVA with Bonferroni correction.

Currently, the molecular mechanisms for increased HBD-2 expression in psoriatic skin are not well defined. Since our findings revealed that NETs were present within psoriatic epidermis in close proximity to keratinocytes which expressed HBD-2, we next investigated whether netting neutrophils from psoriasis patients may induce HBD-2 expression in epidermal keratinocytes. Normal human keratinocytes were obtained from the foreskin of healthy adults, and peripheral blood neutrophils were isolated from 11 healthy controls, 7 eczema patients (general eczema) and 9 psoriasis patients (psoriasis vulgaris). All of the psoriasis patients had PASI score >15, and their peripheral blood neutrophils contained high amounts of NETs as visualized by fluorescence microscopy. Normal human keratinocytes were treated with PMA or DNase I alone, or co-cultured for 16 hours with unstimulated control neutrophils (n = 11), control neutrophils stimulated with PMA (n = 11), control neutrophils treated with both PMA and DNase I (n = 11), unstimulated eczema neutrophils (n = 7), eczema neutrophils stimulated with PMA (n = 7), eczema neutrophils treated with both PMA and DNase I (n = 7), psoriatic neutrophils (n = 9), and psoriatic neutrophils treated with DNase I (n = 9). HBD-2 mRNA expression from keratinocytes was determined by real-time quantitative reverse transcription–polymerase chain reaction (RT-PCR). As shown in Fig. 6a, unstimulated eczema neutrophils did not induce HBD-2 mRNA expression in keratinocytes, compared to healthy control neutrophils. Control or eczema neutrophils stimulated to undergo NETosis with PMA induced HBD-2 mRNA expression in keratinocytes, which was partially attenuated by pre-treatment of NETs with DNase I. Similarly, netting neutrophils from psoriasis patients induced HBD-2 mRNA expression in keratinocytes, which was partially suppressed by DNase I pre-treatment. These results indicate that NETs, including psoriatic NETs, can induce HBD-2 mRNA production in epidermal keratinocytes, and the intact DNA backbone structure of NETs is essential for this process. On the other hand, our results showed that control and eczema neutrophils (with or without PMA stimulation) and psoriatic NETs had no significant effect on LL37 mRNA expression in keratinocytes (Supplementary Figure 2).

### Netting neutrophils from psoriasis patients induced HBD-2 protein secretion from epidermal keratinocytes

Normal human keratinocytes were obtained from the foreskin of healthy adults, and peripheral blood neutrophils were isolated from 7 healthy controls and 7 psoriasis patients. All of the psoriasis patients had PASI score >15, and their peripheral blood neutrophils contained high amounts of NETs as visualized by fluorescence microscopy. Normal human keratinocytes were treated with PMA or DNase I alone, or co-cultured for 24 hours with unstimulated control neutrophils (n = 7), control neutrophils stimulated with PMA (n = 7), control neutrophils treated with both PMA and DNase I (n = 7), psoriatic neutrophils (n = 7), and psoriatic neutrophils treated with DNase I (n = 7). HBD-2 protein levels in keratinocyte culture media was determined by ELISA. As shown in Fig. 6b, control neutrophils stimulated to undergo NETosis with PMA induced HBD-2 protein secretion by keratinocytes, which was partially attenuated by pre-treatment of NETs with DNase I. Similarly, netting neutrophils from psoriasis patients induced HBD-2 protein secretion by keratinocytes, which was partially suppressed by DNase I pre-treatment. These results indicate that NETs, including psoriatic NETs, can induce HBD-2 protein secretion by epidermal keratinocytes.

## Discussion

Psoriasis is a common chronic immune-mediated inflammatory disease, and occurs in around 2% of the population worldwide^1^. Psoriasis may appear at any age, but it is uncommonly seen under the age of 10 years, and is most likely to start between 15–30 years. It is previously regarded as a T helper 1 (Th1) cell-mediated immune disease. More recently, a novel subset of CD4+ T cells named T helper 17 (Th17) cells have been identified, and were shown to play an important role in the development of psoriasis^37^,^38^. Recent studies have also highlighted the role of the innate immune system in the pathogenesis of psoriasis^10^,^11^,^12^. Psoriasis skin lesions may be triggered by physical damage (Koebner phenomenon) and streptococcal throat infections^39^, both of which are associated with activation of innate immunity. In addition, genes involved in innate immunity have been found to be associated with psoriasis susceptibility^40^.

Neutrophils are part of the innate immune system, and are involved in the first line of defense against microbial pathogens. Activated neutrophils may kill microbial pathogens by phagocytosis, or by degranulation with release of bactericidal peptides and reactive oxygen species^41^. More recently, neutrophils have been demonstrated to form neutrophil extracellular traps (NETs), which bind and destroy microbial pathogens (including bacteria, fungi, parasites and viruses) in the extracellular space^19^. Neutrophils release NETs through a distinctive cell death process called “NETosis”. NETs are web-like structures which consist of decondensed DNA associated with histones, antimicrobial peptides and enzymes^20^,^21^,^22^,^23^.

An adverse effect of NETs is that they may act as a source of autoantigens, and may therefore contribute to the development of autoimmune diseases. Kessenbrock *et al.* previously reported that NETosis may play an important role in the pathogenesis of autoimmune small vessel vasculitis by presenting autoantigens (including myeloperoxidase and proteinase-3) to the immune system^25^. NETosis may also play a pathogenic role in SLE, in which NETs may act as a source of autoantigens to induce production of autoantibodies^42^. Recently, increased NETosis has been demonstrated in the blood and tissues of patients with SLE^26^,^27^,^28^. In addition, two different studies have shown that sera from patients with SLE have impaired ability to degrade NETs^24^,^29^.

The role of NETosis in the pathogenesis of psoriasis has not been clearly defined previously. Recently, Lin *et al.* showed that neutrophils and mast cells release IL-17 through formation of extracellular traps in psoriasis skin lesions^18^. Contrary to previous belief that IL-17 was produced by T lymphocytes (Th17 cells), it was demonstrated that the majority of IL-17 was produced by mast cells (mainly by degranulation) and neutrophils (mainly by NETosis) in psoriasis skin lesions. However, the amount of NETotic cells in the peripheral blood of psoriasis patients has not been quantified in previous studies, the ability of psoriasis sera to induce NET formation and NET degradation has not been explored, and the relationship between NETosis and disease severity has not been investigated.

In this study, we demonstrated that patients with psoriasis had higher amount of NETotic cells in their peripheral blood compared to normal healthy controls and patients with eczema, and psoriasis sera showed increased ability to induce NET formation in control neutrophils. The stimuli responsible for inducing NET formation in psoriasis patients are currently unknown. A number of studies have shown that serum levels of certain cytokines, including Th1 cytokines (TNF-α, IFN-γ, IL-12, IL-8) and Th17 cytokines (IL-23, IL-17) are elevated in patients with psoriasis and correlate with disease severity^43^,^44^,^45^. Some of these cytokines, including interferons, TNF-α, IL-8 and IL-17, have been shown to contribute to NET formation in diseases other than psoriasis^19^,^42^,^46^,^47^,^48^. Further investigations are required to identify the specific factors in the serum of psoriasis patients which may be responsible for inducing NETosis.

We also demonstrated that sera from psoriasis patients had normal NET degradation ability. This is in contrast to previous studies showing impaired NET degradation in SLE patients^24^,^29^. The mechanisms responsible for diminished NET breakdown in patients with SLE may involve the presence of circulating DNase I inhibitors or anti-NET protective antibodies which prevent NET degradation. Unlike SLE, psoriasis is not primarily a disease involving the overproduction of various autoantibodies. Hence the sera of psoriasis patients are less likely to contain specific neutralizing DNase I antibodies which may act as DNase I inhibitors, or anti-NET antibodies which may protect NETs from degradation.

Moreover, the amount of NETotic cells in the peripheral blood and the ability of patients’ sera to induce NET formation correlated with psoriasis disease severity. These results suggest that NETs may play a role in the pathophysiology of psoriasis. Previous studies have demonstrated that a critical step in the pathogenesis of psoriasis is the activation of plasmacytoid dendritic cells with interferon-α production^49^, leading to stimulation of CD4+ T cells (including Th1 and Th17 lymphocytes). An important activator of plasmacytoid dendritic cells is LL37-DNA complexes^50^, which have been shown to be a component of NETs in patients with SLE^26^,^27^. In addition, two recent studies have suggested that psoriatic NETs may contain LL37^18^,^51^. Therefore, it is possible that in patients with psoriasis, NETosis may lead to the secretion of LL37-DNA complexes, which may activate plasmacytoid dendritic cells to produce interferon-α, resulting in psoriasis initiation and exacerbation. In patients with psoriasis, LL37 may be produced from two possible sources - keratinocytes or neutrophils. The results of the current study demonstrated that NETs do not induce additional LL37 production from epidermal keratinocytes. Further investigations are required to clarify the relationship between NETosis, LL37 production, and psoriasis development.

Psoriasis skin lesions are characterized by low rates of cutaneous bacterial, fungal and viral infections^31^. This has been attributed to the increased expression of various antimicrobial peptides in psoriasis plaques^32^. HBD-2 is an antimicrobial peptide expressed by epithelial cells which demonstrates a broad spectrum of antimicrobial activity^52^. Recent studies have shown that increased β-defensin genomic copy number is associated with the risk of developing psoriasis^53^. High expression of HBD-2 can be found in psoriasis skin lesions^33^,^34^,^35^, localized to the upper layers of the epidermis and the stratum corneum^54^,^55^. In contrast, HBD-2 expression is negligible in normal skin and low in skin lesions of eczema^33^,^34^,^35^,^36^. Due to its marked differential expression in psoriasis plaques compared to non-psoriatic skin, HBD-2 has been regarded as the strongest and most psoriasis-specific protein^34^. In addition, serum HBD-2 levels have been found to correlate with psoriasis disease activity^35^. In this study, we confirmed by immunohistochemical staining that HBD-2 peptide is highly expressed in psoriasis lesional skin, but expressed at negligible or low levels in normal skin, eczema lesions, and psoriasis nonlesional skin.

In this study, we showed by confocal microscopy that neutrophils undergoing NETosis were frequently present in the epidermis of psoriasis plaques, and immunohistochemical analysis demonstrated strong epidermal expression of HBD-2 peptide in the majority of psoriasis skin lesions. Since NETs were found within psoriatic epidermis in close proximity to epidermal keratinocytes which expressed HBD-2, we investigated whether netting neutrophils from psoriasis patients may induce HBD-2 production from keratinocytes. We demonstrated that NETs from psoriasis patients strongly induced HBD-2 mRNA expression and protein secretion in epidermal keratinocytes, and the intact DNA backbone structure of NETs is essential for this process. Therefore, NET-induced HBD-2 production may provide a novel mechanism for the decreased susceptibility of psoriasis plaques to microbial infections. Previously, HBD-2 has also been demonstrated to be chemotactic for dendritic cells, T cells and neutrophils^56^,^57^. This interplay between innate and adaptive immunity may further contribute to the antimicrobial defense mechanisms of psoriasis.

A previous study by Harder *et al.* found increased expression and secretion of various antimicrobial peptides (including ribonuclease 7, psoriasin, HBD-2 and HBD-3) in skin lesions of atopic dermatitis, psoriasis and following skin barrier disruption. Although the levels of these antimicrobial peptides were increased in atopic dermatitis skin lesions compared to normal skin, they were significantly lower compared to skin lesions of psoriasis^58^. The results of the current study demonstrated that in patients with psoriasis, NETosis induced HBD-2 expression in keratinocytes. On the other hand, the amount of NETs was not found to be significantly elevated in patients with atopic dermatitis included in this study. Therefore, enhanced NETosis may contribute to the higher antimicrobial peptide expression frequently observed on psoriatic skin as compared to atopic skin. However, it should be noted that atopic dermatitis is a complex and heterogeneous disease, and differences in antimicrobial peptide expression were found between atopic skin lesions at different stages^58^. Further investigations are required to determine whether NETosis may contribute to antimicrobial peptide expression in a subset of patients with atopic dermatitis.

Our findings may have a number of clinical implications. Firstly, the increased formation of NETs in patients with psoriasis and its correlation with disease severity indicate that the degree of NETosis may be useful as a biomarker for psoriasis disease severity. Currently, PASI score is the most commonly used severity assessment tool for psoriasis. However, this clinical scoring criteria is based partly on subjective judgment, semi-quantitative in nature, time-consuming to perform, and its accuracy may be limited by interrater and intrarater variability^59^. The findings of this study indicate that the amount of NETotic cells in the peripheral blood may complement the PASI score in the assessment of psoriasis disease severity. Secondly, investigating the role of NETosis in psoriasis may lead to the development of new therapeutic strategies in the future. More specifically, further studies are required to determine whether inhibition of NETosis, for example with reactive oxygen species scavengers, nicotinamide adenine dinucleotide phosphate (NADPH) oxidase inhibitors or dimethylfumarate^16^, may have therapeutic potential in patients with psoriasis.

In conclusion, this study demonstrates that patients with psoriasis have higher amount of NETotic cells in their peripheral blood compared to healthy controls and patients with eczema, and the amount of NETs correlates with psoriasis disease severity. Compared to healthy controls, psoriasis sera have increased ability to induce NET formation in control neutrophils, but normal NET degradation ability. Moreover, neutrophils undergoing NETosis are frequently observed in the epidermis of psoriatic plaques. In addition, psoriatic netting neutrophils are strong inducers of HBD-2 expression in epidermal keratinocytes, and the intact DNA backbone structure of NETs is essential for this process. These findings offer new insights into the significance of NETosis in the pathophysiology of psoriasis. Furthermore, NET-induced HBD-2 production may provide a novel mechanism for the decreased susceptibility of psoriasis plaques to microbial infections.

## Methods

### Recruitment of patients

Forty-eight patients with psoriasis and 35 patients with eczema (including contact dermatitis, atopic dermatitis and general eczema) were enrolled from the Department of Dermatology, Kaohsiung Medical University Hospital and Ministry of Health and Welfare Pingtung Hospital. Additionally, 48 healthy controls individually matched by age (within 5 years) and sex to psoriasis cases were recruited by advertisement. Peripheral venous blood samples were taken from patients and healthy controls. Clinical data on psoriasis subtype (psoriasis vulgaris, guttate psoriasis, erythrodermic psoriasis), presence of psoriatic arthritis, disease severity, and treatment for psoriasis (topical therapy, phototherapy, immunosuppressive medications, biological agents) were collected at the time of blood sampling. Psoriasis disease severity in each patient was evaluated using the PASI score, which assesses the severity of psoriasis skin lesions and the area affected. The study was approved by the ethics committee of Kaohsiung Medical University Hospital and conducted according to the Declaration of Helsinki. Written informed consent was obtained from all patients and healthy controls prior to participation in this study.

### Isolation of peripheral blood neutrophils

Human neutrophils were isolated from peripheral venous blood by the method of density gradient separation as described previously^60^. Whole blood from patients with psoriasis, patients with eczema and healthy controls was collected in tubes with sodium heparin as anticoagulant. Neutrophil isolation was performed immediately after blood collection. Seven milliliters of whole blood was layered on top of 7 ml of Histopaque 1119 (Sigma-Aldrich, St. Louis, MO, USA) in a 15 mL conical centrifugation tube. The tube was centrifuged at 800 g for 20 minutes at room temperature. The upper layer containing lymphocytes and monocytes was removed and the lower polymorphonuclear cell layer (the layer directly above the red blood cell layer) was collected with a plastic Pasteur pipette. The polymorphonuclear cell layer was washed in phosphate buffered saline (PBS), centrifuged at 300 g for 10 minutes at room temperature, and the cell pellet was collected.

Percoll gradients (Sigma-Aldrich) were prepared in a 15 mL centrifugation tube, by placing different concentrations of isotonic Percoll solutions on top of each other (2 mL each of 85%, 80%, 75%, 70% and 65%), beginning with 85% solution at the bottom. The polymorphonuclear cell pellet was resuspended in 2 ml PBS and placed on top of the Percoll gradient. The gradients were centrifuged at 800 g for 20 minutes at room temperature, and the interphase layer between the 70% and 75% Percoll layers was collected in a 15 mL tube. The tube was filled with PBS and centrifuged at 300 g for 10 minutes at room temperature. The cells were then resuspended in RPMI medium (Invitrogen Carlsbad, CA, USA) with 10 mM HEPES (Invitrogen) and 0.5% human serum albumin (Sigma-Aldrich).

### Visual quantification of NETosis by fluorescence microscopy

Neutrophils were seeded in RPMI medium with 0.5% human serum albumin in 6-well plates, and were incubated at 37 °C for 30 minutes to enable them to adhere. 200 nM of Sytox Green (Invitrogen) was added to each well, and the plates were incubated in the dark for 10 minutes at room temperature. Sytox Green is a cell-impermeable fluorescent DNA dye which can stain extracellular DNA structures such as NETs. Images of each well were then taken using a Nikon ECLIPSE Ti inverted fluorescence microscope with NIS-Elements F3.2 software (Nikon, Tokyo, Japan). The fluorescence images and their corresponding phase contrast images were captured for at least ten random fields at 100× magnification. The total number of cells was counted using the phase contrast images. Using the fluorescence images, the number of cells undergoing NETosis was counted. A NETotic cell has a cloud-like appearance with decondensed nuclei, has a larger area than an unstimulated neutrophil, and shows extracellular web-like DNA strands. Visual quantification of NETosis was performed by two independent investigators. The number of cells undergoing NETosis was divided by the total number of neutrophils to obtain the percentage of NETotic cells.

### NET formation assay

Sera were collected from peripheral blood samples of psoriasis patients and healthy controls following centrifugation at 1700 g for 10 minutes at room temperature, and stored at −80 °C until further use. On the day of experimentation, peripheral blood neutrophils were isolated from healthy controls using density gradient separation as described above. The control neutrophils were resuspended at a density of 10^6^ cells/mL in RPMI medium with 0.5% human serum albumin in 12-well plates. The unstimulated control neutrophils were then incubated with 10% serum from psoriasis patients or healthy controls at 37 °C for 2 hours. To detect NETotic cells, 200 nM of Sytox Green was added to each well, and visual quantification of NETosis was performed by fluorescence microscopy as described above.

### NET degradation assay

Freshly isolated control neutrophils from a healthy donor were seeded at a density of 10^6^ cells/mL in RPMI medium with 0.5% human serum albumin in 24-well plates. The neutrophils were incubated at 37 °C for 30 minutes to allow them to adhere. Control neutrophils were stimulated by adding 25 nM of PMA (Sigma-Aldrich) for 2 hours to induce NETosis. The morphology of the cells was evaluated using fluorescence microscopy to confirm induction of NETs.

Wells containing NETs were incubated with 10% serum from psoriasis patients or healthy controls at 37 °C for 6 hours. EDTA (2 mM) was added to stop nuclease activity, and the culture supernatants were collected and transferred to a black 96-well plate. Control sera treated with the DNase I inhibitor G-actin was included as a positive control. The fluorescent DNA dye PicoGreen (1:200 dilution; Invitrogen) was added, and the DNA content was assessed using a fluorescence spectrophotometer (Bio-Tek, Winooski, VT, USA) with a 485 nm excitation filter and 528 nm emission filter. The results were reported as DNA fluorescence. For analysis of psoriasis sera, the amount of NET degradation by the sera of healthy controls was regarded as 100%.

### Visualization of NETosis in skin specimens by confocal microscopy

Twenty cases of psoriasis skin tissue specimens and 20 cases of skin eczema specimens were retrospectively acquired from our pathology department. Five micrometer paraffin-embedded skin tissue sections were mounted on glass slides, deparaffinized in xylene, and rehydrated with distilled H_2_O through a graded series of alcohols. Following antigen retrieval with citrate buffer, the slides were incubated with 3% H_2_O_2_ for 10 minutes, and then blocked in 1% bovine serum albumin for 1 hour. The slides were first stained with rabbit neutrophil elastase polyclonal antibody (Abcam, Cambridge, United Kingdom) overnight at 4 °C, followed by Alexa Fluor 568-conjugated goat anti-rabbit secondary antibody (Invitrogen) for 1 hour. The slides were then stained with rabbit histone H2A monoclonal antibody (Cell Signaling Technology, Danvers, MA, USA) for 4 hours at room temperature, followed by Alexa Fluor 488-conjugated goat anti-rabbit secondary antibody (Invitrogen) for 1 hour. Finally, the slides were stained with the DNA stain DAPI (Invitrogen). The slides were subsequently mounted using ProLong Gold antifade reagent (Invitrogen), and visualized with a confocal microscope (FV-1000; Olympus, Tokyo, Japan). NETs were identified as extracellular strandlike DNA structures which co-localized with histones and neutrophil elastase.

### Immunohistochemical staining of tissue specimens

Immunohistochemical staining of skin tissue specimens for HBD-2 was performed for 28 cases of psoriasis lesional skin, 10 cases of psoriasis nonlesional skin, 15 cases of eczema skin (general eczema), and 10 cases of normal skin. Three micrometer paraffin-embedded tissue sections were deparaffinized in xylene and rehydrated. Antigen retrieval was conducted by immersing slides in citrate buffer. Prior to staining, endogenous peroxidase was inhibited by 3% H_2_O_2_, and non-specific protein binding was blocked by incubating the slides in 1% bovine serum albumin. The slides were then incubated with HBD-2 antibody (1:500 dilution; Santa Cruz Biotechnology, Dallas, TX, USA) at 4 °C overnight, followed by biotinylated secondary antibody (Dako Corporation, Carpinteria, CA, USA) for 30 minutes at room temperature. Immunoreactivity was detected with 3,30-diaminobenzidine substrate-chromogen solution (Dako), and the slides were counterstained with hematoxylin. Images were acquired using an Olympus BX53 microscope with DP70 digital camera system and DP Controller software (Olympus, Tokyo, Japan). Immunohistochemical staining was assessed by two independent investigators.

### Keratinocyte culture

Human keratinocytes were obtained from normal adult foreskin following routine circumcision surgery. Skin specimens were cleaned of adipose tissue, cut into small fragments and incubated in Dispase II at 37 °C for 30 minutes. The epidermal layer containing keratinocytes was carefully separated from the dermis using fine forceps, and 0.25% trypsin–EDTA was added for 12 minutes at 37 °C to release epidermal keratinocytes. The trypsin reactivity was neutralized by addition of 10% FBS. Following centrifugation (190 g for 10 minutes), the cell pellet was collected. The keratinocytes were cultured in serum-free keratinocyte-SFM medium (Life Technologies, Carlsbad, CA, USA) supplemented with 5 ng/ml human recombinant epidermal growth factor and 25 μg/ml bovine pituitary extract (Life Technologies). Cells were incubated at 37 °C with 5% CO_2_ in a humidified atmosphere. Keratinocytes of passage 3 were used for experiments.

### Determination of HBD-2 and LL37 mRNA expression in keratinocytes

Peripheral blood neutrophils were isolated from healthy controls, eczema patients and psoriasis patients. Following various treatments, the neutrophils were co-cultured for 16 hours with normal human keratinocytes. Quantification of HBD-2 and LL37 mRNA expression from keratinocytes was performed by real-time quantitative RT-PCR. Briefly, total RNA was extracted from keratinocytes using TRIzol Reagent (Invitrogen). RNA was reverse transcribed into cDNA using a cDNA synthesis kit which contains the Moloney murine leukemia virus (M-MLV) reverse transcriptase (GoScript^TM^ Reverse Transcriptase, Promega, Madison, WI, USA). Specific primers for HBD-2 and LL37 were constructed using Beacon Designer 5 (Premier Biosoft, Palo Alto, CA, USA). The PCR primer sequences were as follows: 5′-CCAGCCATCAGCCATGAGGGT-3′ (sense) and 5′-GGAGCCCTTTCTGAATCCGCA-3′ (antisense) for HBD-2 (DEFB4A); 5′-GCTAACCTCTACCGCCTCCTG-3′ (sense) and 5′-CCGTCCTTCTTGAAGTCACAATCC-3′ (antisense) for LL37 (cathelicidin antimicrobial peptide); 5′-CGCTGAGTACGTCGTGGAGTC-3′ (sense) and 5′-GAGGCATTGCTGATGATCTTGAGG-3′ (antisense) for GAPDH. Real-time PCR was performed to amplify the cDNA samples for 40 cycles with the Applied Biosystems 7500 Real-Time PCR System (Applied Biosystems, Foster City, CA, USA), and gene-specific PCR products were quantified using SYBR Green dye (Applied Biosystems). The PCR cycling parameters included an initial activation step for 10 minutes at 95 °C, followed by 40 cycles consisting of a denaturation step for 15 seconds at 95 °C and a combined annealing⁄extension step for 1 minute at 60 °C. The cycle thresholds (Ct) for HBD-2 and LL37 in each sample were determined. Relative HBD-2 mRNA and LL37 gene expression levels were calculated using the 2^−ΔΔCt^ method, and results were normalized to the internal control GAPDH. Melting curve analyses were carried out to confirm specificity of amplified gene products. Real-time PCRs were performed in triplicate for each sample.

### Determination of HBD-2 protein secretion by keratinocytes

Peripheral blood neutrophils were isolated from 7 healthy controls and 7 psoriasis patients. Following various treatments, the neutrophils were co-cultured for 24 hours with normal human keratinocytes. For quantification of HBD-2 protein secretion by keratinocytes, cell culture media was collected and HBD-2 protein level was measured by a commercially acquired HBD-2 ELISA kit (Peprotech, Rocky Hill, NJ, USA) in accordance with the manufacturer’s instructions.

### Statistical analysis

The data were presented as mean ± standard deviation (SD). Fisher’s exact test was used for analysis of categorical data. The two sample t-test was used to compare continuous data from two groups. For comparisons of continuous data from multiple groups, one-way analysis of variance (ANOVA) with Bonferroni correction was performed. Correlation of PASI score with NET amount and NET formation was determined using Spearman’s correlation test. Statistical analysis was assessed using SPSS software (SPSS Inc., Chicago, IL, USA). All statistical tests were two-sided. Statistical significance was defined at *P* < 0.05.

## Additional Information

**How to cite this article**: Hu, S. C.-S. *et al.* Neutrophil extracellular trap formation is increased in psoriasis and induces human β-defensin-2 production in epidermal keratinocytes. *Sci. Rep.* **6**, 31119; doi: 10.1038/srep31119 (2016).

## Supplementary Material

Supplementary Information

## Figures and Tables

**Table 1:** Association between amount of NETotic cells in the peripheral blood (low versus high NET amount) and clinical parameters in patients with psoriasis (n = 48).

Clinical parameter	Low NET amount (n = 23)^a^	High NET amount (n = 25)	*P* value^b^
Age	47.96 ± 14.45	47.20 ± 16.53	0.867
Sex			0.777
Male (n = 28)	14 cases	14 cases	
Female (n = 20)	9 cases	11 cases	
Clinical subtype			0.740
Psoriasis vulgaris (n = 44)	22 cases	22 cases	
Guttate psoriasis (n = 2)	1 case	1 case	
Erythrodermic psoriasis (n = 2)	0 cases	2 cases	
Psoriatic arthritis			0.668
Yes (n = 6)	2 cases	4 cases	
No (n = 42)	21 cases	21 cases	
PASI score	14.37 ± 7.12	22.71 ± 7.94	**<0.001**
Treatment^c^			
No treatment (n = 7)	3 cases	4 cases	1.000
Topical therapy (n = 34)	17 cases	17 cases	0.756
Phototherapy (n = 28)	13 cases	15 cases	1.000
Methotrexate (n = 6)	2 cases	4 cases	0.668
Cyclosporine (n = 4)	2 cases	2 cases	1.000
Biological agent (n = 3)	2 cases	1 case	0.601

^a^Patients with psoriasis were categorized into the low NET amount group or high NET amount group using the mean amount of NETotic cells in the peripheral blood (11.53%) as the dividing point.

^b^The *P* values for age and PASI score were determined using the two sample t-test. For other variables (sex, clinical subtype, psoriatic arthritis, treatment), the *P* values were determined using Fisher’s exact test.

^c^For analysis of treatment, the numbers of patients who had or had not received a particular form of treatment were compared.
