# Supplementary material for: Neutrophil extracellular trap formation is increased in psoriasis and induces human β-defensin-2 production in epidermal keratinocytes
Source: Sci Rep. 2016 Aug 5;6:31119. doi: 10.1038/srep31119 (PMC4974609; doi:10.1038/srep31119)
Supplement: Supplementary Information [file srep31119-s1.pdf]

Neutrophil extracellular trap formation is increased in psoriasis and induces human β-defensin-2 production in epidermal keratinocytes

Stephen Chu-Sung Hu, MBBS, M.Phil [1,2]
Hsin-Su Yu, MD, PhD [1,2,3]
Feng-Lin Yen, PhD [4]
Chi-Ling Lin, MD [2,5]
Gwo-Shing Chen, MD, PhD [1,2]
Cheng-Che E. Lan, MD, PhD [1,2,*]

[1] Department of Dermatology, College of Medicine, Kaohsiung Medical University, Kaohsiung, Taiwan
[2] Department of Dermatology, Kaohsiung Medical University Hospital, Kaohsiung, Taiwan
[3] National Institute of Environmental Health Sciences, National Health Research Institutes, Taipei, Taiwan
[4] College of Pharmacy, Kaohsiung Medical University, Kaohsiung, Taiwan
[5] Department of Dermatology, Kaohsiung Municipal Hsiao-Kang Hospital, Kaohsiung, Taiwan

* Correspondence: Cheng-Che E. Lan, Department of Dermatology, Kaohsiung Medical University Hospital, No. 100, Tzyou 1st Road, Kaohsiung 807, Taiwan.
Tel: +886 7 3121101 Fax: +886 7 3118894 E-mail: laneric@cc.kmu.edu.tw

Supplementary Figure 1. (a, b, c) Confocal microscopic images of eczema skin tissue specimens. Lymphocytes (white arrows) and neutrophils (yellow arrows) were sometimes seen in the epidermis next to keratinocytes (green arrows). However, NETs were not observed (absence of strandlike DNA structures which co-localized with histones and neutrophil elastase) in eczema skin lesions. Skin specimens were stained for DNA (DAPI, blue), histones (red) and neutrophil elastase (NE, green). Scale bars = 10 μm.

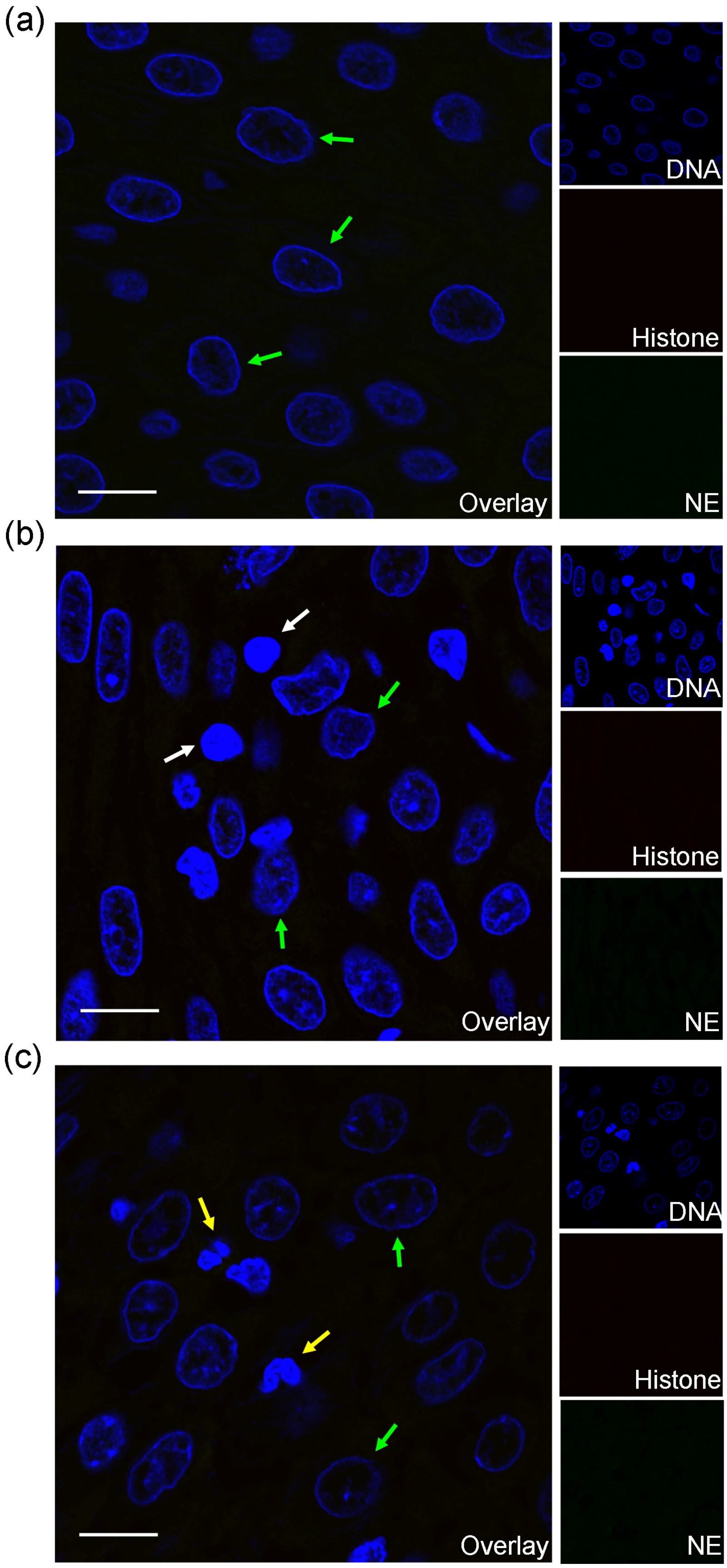

Supplementary Figure 2. Netting neutrophils had no significant effect on LL37 mRNA expression in epidermal keratinocytes. Normal human keratinocytes were obtained from adult foreskin, and peripheral blood neutrophils were isolated from 7 healthy controls, 7 eczema patients and 7 psoriasis patients. Normal human keratinocytes were co-cultured for 16 hours with unstimulated control neutrophils (n = 7), control neutrophils stimulated with PMA (n = 7), unstimulated eczema neutrophils (n = 7), eczema neutrophils stimulated with PMA (n = 7), and psoriatic neutrophils (n = 7). LL37 mRNA expression from keratinocytes was determined by real-time quantitative RT-PCR. 1,25-Dihydroxyvitamin D_3 (10^{-8} M) was used as a positive control to induce LL37 expression in keratinocytes.

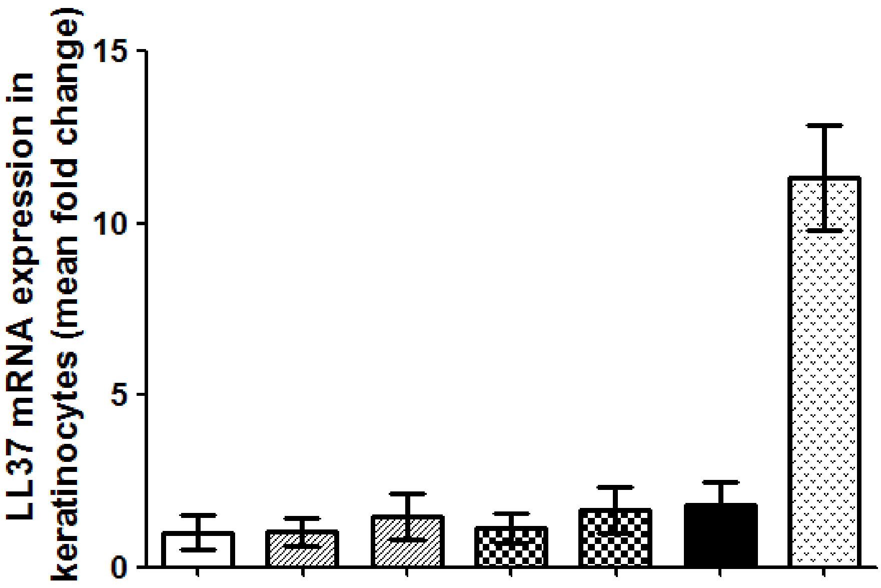

Control neutrophils	–	+	+	–	–	–	–
Eczema neutrophils	–	–	–	+	+	–	–
Psoriatic NETs	–	–	–	–	–	+	–
PMA	–	–	+	–	+	–	–
Vitamin D3	–	–	–	–	–	–	+
